# Gamification of health professions education: a systematic review

**DOI:** 10.1007/s10459-020-10000-3

**Published:** 2020-10-31

**Authors:** A. E. J. van Gaalen, J. Brouwer, J. Schönrock-Adema, T. Bouwkamp-Timmer, A. D. C. Jaarsma, J. R. Georgiadis

**Affiliations:** 1 Department of Biomedical Sciences of Cells and Systems, Section Anatomy & Medical Physiology, University Medical Center Groningen, University of Groningen, Groningen, The Netherlands; 2 Faculty Behavioural and Social Sciences, Department of Educational Sciences, University of Groningen, Groningen, The Netherlands; 3 Center for Education Development and Research in Health Professions (CEDAR), LEARN, University Medical Center Groningen, University of Groningen, 713AV Groningen, The Netherlands

**Keywords:** Gamification, Serious games, Simulation, Game-based learning, E-learning, Computers, New technology, Instructional methods

## Abstract

Gamification refers to using game attributes in a non-gaming context. Health professions educators increasingly turn to gamification to optimize students’ learning outcomes. However, little is known about the concept of gamification and its possible working mechanisms. This review focused on empirical evidence for the effectiveness of gamification approaches and theoretical rationales for applying the chosen game attributes. We systematically searched multiple databases, and included all empirical studies evaluating the use of game attributes in health professions education. Of 5044 articles initially identified, 44 met the inclusion criteria. Negative outcomes for using gamification were not reported. Almost all studies included assessment attributes (n = 40), mostly in combination with conflict/challenge attributes (n = 27). Eight studies revealed that this specific combination had increased the use of the learning material, sometimes leading to improved learning outcomes. A relatively small number of studies was performed to explain mechanisms underlying the use of game attributes (n = 7). Our findings suggest that it is possible to improve learning outcomes in health professions education by using gamification, especially when employing game attributes that improve learning behaviours and attitudes towards learning. However, most studies lacked well-defined control groups and did not apply and/or report theory to understand underlying processes. Future research should clarify mechanisms underlying gamified educational interventions and explore theories that could explain the effects of these interventions on learning outcomes, using well-defined control groups, in a longitudinal way. In doing so, we can build on existing theories and gain a practical and comprehensive understanding of how to select the right game elements for the right educational context and the right type of student.

## Introduction

Gamification is rapidly becoming a trend in health professions education. This is at least suggested by the number of peer-reviewed scientific publications on gamification in this field, which has increased almost tenfold over the past 5 years. At the same time, there seems to be little shared understanding of what constitutes gamification and how this concept differs from other, related concepts. Furthermore, according to business and education literature, there is still no clear understanding of when and why gamification can be an appropriate learning and instructional tool (Dicheva et al. 2015; Landers et al. 2015). The purpose of this systematic review was to provide a comprehensive overview of the use and effectiveness of gamification in health professions education and to add to the existing research on gamification in several ways. To this end, we first clearly and carefully distinguished gamification studies from studies investigating other types of game-based learning. Then, we summarized the contexts in which the gamification interventions took place and their underlying theories. Finally, we analysed the effects of individual game elements by using a conceptual framework that was originally developed by Landers et al. to structure game elements in other, non-educational domains (Landers 2014).

### What is (or is not) gamification?

The use of game design elements to enhance academic performance (e.g., learning attitudes, learning behaviours and learning outcomes) is known as gamification or ‘gamified learning’ (Deterding et al. 2011). Due to the rapidly growing body of information and proliferation of different types of game-based learning (e.g. serious games), authors tend to use different terms for the same concept, or the same term for different concepts (for examples see Amer et al. 2011; Borro-Escribano et al. 2013; Chan et al. 2012; Chen et al. 2015; Frederick et al. 2011; Gerard et al. 2018; Lim and Seet 2008; Stanley and Latimer 2011; Webb and Henderson 2017). In part, this indiscriminate use of terms may be caused by the fact that in the literature of play and gaming there is neither consensus on what a ‘game’ is conceptually (Arjoranta 2014; Caillois 1961; Huizinga 1955; Ramsey 1923; Salen and Zimmerman 2004; Suits 1978; Sutton-Smith 2000), nor on what the essential elements of a game are (Bedwell et al. 2012; Klabbers 2009).

Since there was a lack of uniformity in the definitions of the main forms of game-based learning—gamification, serious games, and simulations—we chose well-known, academically accepted definitions to distinguish among the three concepts that guided the search strategy and enabled selection of articles relevant for this systematic review. First, although various definitions of *gamification* can be found in various fields of literature such as business, education and information technology (Blohm and Leimeister 2013; Burke 2014; Burkey et al. 2013; De Sousa Borges et al. 2014; Domínguez et al. 2013; Gaggioli 2016; Hamari et al. 2014; Hsu et al. 2013; Huotari and Hamari 2012, 2017; Kumar 2013; Muntean 2011; Perrotta et al. 2013; Robson et al. 2015; Simões et al. 2013; Warmelink et al. 2018; Zuk 2012), a commonly applied definition is that of Deterding et al. (De-Marcos et al. 2014; Deterding et al. 2011; Dicheva et al. 2015; Lister et al. 2014; Mekler et al. 2017; Sailer et al. 2017; Seaborn and Fels 2015). He and his colleagues define gamification as the use of game elements (e.g. points, leader boards, prizes) in non-gaming contexts (Deterding et al. 2011). This implies that, even though game elements are used in a certain context (such as education), there should be *no intention of creating a game.* Second, this intention is different from the intention in serious games, which are defined as *games* (in their various forms) in which education is the primary goal, rather than entertainment (Susi et al. 2007). Serious games address real-world topics in a gameplay context. In contrast to gamification, serious game designers’ *intention is to create a game.* Therefore, the characteristic difference between gamification and serious games lies in their design intention. Third, a simulation can be defined as a situation in which a particular set of conditions is created artificially in order to study or experience something that could exist in reality (Oxford English Dictionary 2017). Simulations provide instant feedback on performance, which is delivered as accurate and realistic as possible in a safe environment (Alessi 1988; Vallverdú 2014). Simulations do not need game elements like a scoring system and a win/lose condition. However, game-design techniques and solutions can be employed to create the simulated reality and the experience of something real (Alessi 1988; Jacobs and Dempsey 1993). Simulations are therefore best seen as learning activities that necessarily carry some game intention, but do not use game elements. By explicitly adding the designers’ intentions to the chosen definitions for these three types of game-based learning, we established criteria for distinguishing between them and guiding study inclusion and analysis in this systematic review.

### Game elements and game attributes

In academic literature, various game elements have been proposed to improve the learning experience in gamification, e.g. rewards, leader boards and social elements (Petrucci et al. 2015; Van Dongen et al. 2008). In addition, grey literature also lists vast amounts of different types of game elements (Marczewski 2017), which—though lacking an academic framework or basis—have been used in previous research (Wells 2018). Different terminology is used for the same type of game-elements; for example, badges (Davidson and Candy 2016), donuts (Fleiszer et al. 1997) and iPads (Kerfoot and Kissane 2014) are all types of rewards. In a recent systematic review on serious games (and gamification), Gorbanev et al. (2018) ascertained a lack of consensus regarding terminology used in games and welcomed any effort to reduce this terminological variety. Therefore, to make the results of gamification research in health professions education more comprehensive and transferable with regard to the game elements involved, we applied a conceptual framework of aggregated game elements that was originally proposed by Bedwell et al. (2012) and Wilson et al. (2009), and later modified by Landers (2014) (Table 2; “Appendix”). This framework posits that all existing game elements can be described and structured into nine attributes, while avoiding significant overlap between these attributes (Bedwell et al. 2012). The following game attributes are included in the framework: action language, assessment, conflict/challenge, control, environment, game fiction, human interaction, immersion and rule/goals. Instead of only focusing on specific game elements, we chose to use this framework to identify whether there is a *class* of game elements that hold the highest promise of improving health professions education. In doing so, we added a new perspective to existing reviews on gamification in higher (Caponetto et al. 2014; De Sousa Borges et al. 2014; Dichev and Dicheva 2017; Dicheva et al. 2015; Faiella and Ricciardi 2015; Nah et al. 2014; Subhash and Cudney 2018) and health professions education (Gentry et al. 2016; McCoy et al. 2016). In addition, we attempted to uncover the theory underpinning the gamified interventions reported in this systematic review. In doing so, we responded to the call for more theory-driven medical education research (Bligh and Parsell 1999; Bligh and Prideaux 2002; Cook et al. 2008), which we felt should also apply to research on game-based learning in general and gamification in particular (Caponetto et al. 2014; De Sousa Borges et al. 2014; Faiella and Ricciardi 2015; Gentry et al. 2016; McCoy et al. 2016; Nah et al. 2014).

This study, therefore, was intended to contribute to the literature (including existing systematic reviews) in several ways: (1) by creating a sharper distinction between gamification and other forms of game-based learning; (2) by using a more generic way to structure game elements; and (3) by responding to the call for more theory-driven medical education research.

In sum, this systematic review aimed to provide teachers and researchers with a comprehensive overview of the current state of gamification in health professions education, with a particular focus on the effects of gamification elements on learning, the underlying mechanisms and considerations for future research.

We formulated five principal research questions that guided this systematic review:

1. What are the contexts in which game elements are used in health sciences education?
2. What game elements are tested and what attributes do they represent?
3. Is there empirical evidence for the effectiveness of gamified learning in health professions education?
4. What is the quality of existing research on gamified learning in health professions education?
5. What is the theoretical rationale for implementing gamified learning in health professions education?

## Methods

We conducted a systematic review in accordance with the guidelines of the Associations for Medical Education in Europe (AMEE) (Sharma et al. 2015).

### Search strategy

We systematically searched the literature for publications on the use of game elements in health professions education. First, we consulted two information specialists with expertise in systematic reviews to assist in developing the search strategy. Together, we identified keywords, key phrases, synonyms and alternative keywords for gamification as well as game elements and attributes that were derived from a list of 52 game elements and Landers’ framework. Some of the 52 game elements and attributes were omitted from the search as they were too broad and generated a huge amount of hits (e.g., ‘environment’, ‘scarcity’, ‘consequences’), irrelevant hits or as they did not generate any hits (e.g. ‘Easter eggs’). Based on these main keywords we formulated the search strategy for PubMed.

The first author (AvG) translated the PubMed search strategy for use in other databases and then systematically searched eight databases: Academic Search Premier; CINAHL; EMBASE; ERIC; Psychology and Behavior Sciences Collection; PsychINFO, PubMed and the Cochrane Library. The search was performed in April 2018. We used the following search terms: (gamif* OR gameplay* OR game OR games OR gamelike OR gamebased OR gaming OR videogam* OR edugam* OR flow-theor* OR “social network*” OR scoreboard* OR leveling OR levelling OR contest OR contests OR badgification) AND (medical educat* OR medical train* OR medical field training OR medical school* OR medical Intern* OR medical residen* OR medical student* OR dental student* OR nursing student* OR pharmacy student* OR veterinary student* OR clinical education* OR clinical train* OR clinical Intern* OR clinical residen* OR clinical clerk* OR teaching round* OR dental education* OR pharmacy education* OR pharmacy residen* OR nursing education* OR paramedics education* OR paramedic education* OR paramedical education* OR physiotherapy education* OR physiotherapist education* OR emergency medical services educat* OR curricul* OR veterinary education OR allied health personnel).

### Inclusion criteria

We included peer-reviewed journal articles on the use of gamification or game elements in education for (future) health professionals. We defined health professionals as individuals oriented towards providing curative, preventive and rehabilitative health care for humans as well as animals (e.g. individuals working in the fields of medicine, nursery, pharmacology and veterinary medicine). Real-life contexts (e.g. lectures and practicals) as well as digital contexts (e.g. mobile applications or computer software) were eligible for inclusion if they incorporated gamified learning to improve (future) health professionals’ (bio)medical knowledge or skills. We included quantitative as well as mixed-method studies.

### Exclusion criteria

We excluded articles that (a) only described the development of gamified learning activities in educational contexts without reporting the effects of their interventions, (b) only focused on qualitative data, (c) focused on serious games, (d) focused on patient education, (e) focused on simulations, except when the focus was on the effects of gamification in simulations (gamified simulations), (f) described adapted environments such as game-shows (e.g., “jeopardy” or “who wants to be a millionaire”) and board-games (e.g., “monopoly” or “trivial pursuit”) which we considered to be game contexts, and (g) were not written in Dutch or English.

Although the term “gamification” has been used since 2008 (Deterding et al. 2011; Szyma 2014), we did not set a timeframe for our search since individual game elements were used in a non-game context long before the term “gamification” was coined and appeared in scientific literature.

### Study selection

After retrieving the search results from different databases, AvG removed the duplicates and uploaded the remaining articles to Rayyan, a mobile and web-based application developed for systematic reviews (Ouzzani et al. 2016). Then, AvG and JB independently screened all titles and abstracts for preliminary eligibility. In case of uncertainty, the articles in question were retained. Subsequently, AvG read the full text of all retained articles to determine eligibility for inclusion in this systematic review. In case of uncertainty, articles were marked for discussion and independently screened by all researchers. To ensure consistency in the application of selection criteria, we undertook double screening on a 10% random sample of the excluded articles as a form of triangulation. The researchers met at a regular basis to discuss challenges, uncertainties and conflicts with respect to article selection. Disagreement between researchers was resolved by discussion.

In addition, we hand-searched the reference lists of included articles and citations for additional articles.

### Data extraction and quality assessment processes

We extracted the following data from the included articles using the extraction methods described in the AMEE guideline (Sharma et al. 2015):

1. General information (e.g., author, title and publication year), participant characteristics (including demographics and sample size) and characteristics of the educational content (including topic and the context in which the topic is presented, e.g. digital or analogue and type of study or profession);
2. Intervention (type of game element(s) and game attributes used);
3. Study outcomes (including satisfaction, attitudes, perceptions, opinions, knowledge, behaviour and patient outcomes);
4. Study quality (see below);
5. Theoretical frameworks used to design or evaluate gamified educational programs.

We used the Medical Education Research Study Quality Instrument (MERSQI) to measure the methodological quality of the selected studies (Reed et al. 2007). MERSQI is designed for measuring the quality of experimental, quasi-experimental and observational studies and consists of ten items covering six domains (study design, sampling, type of data, validity of evaluation instrument, data analysis, and outcomes). The maximum score for each domain is three. Five domains have a minimum score of one, resulting in a range of 5–18 points. We calculated individual total MERSQI scores, mean scores and standard deviations.

To provide a clear overview of the current state of studies on gamification in health sciences education, we used the framework for classifying the purposes of research in medical education proposed by Cook et al. (2008). They distinguished studies as focusing on description, justification and clarification. Description studies make no comparison, focus on observation and describe what was done. Justification studies make comparisons between interventions, generally lack or do not present a conceptual framework or a predictive model and describe whether the new intervention worked. Clarification studies apply a theoretical framework to understand and possibly explain the processes underlying the observed effects, describe why and how interventions (i.e. gamification) work and illuminate paths of future directions (Cook et al. 2008).

We classified all included studies as descriptive, justification or clarification.

## Results

**Fig. 1:**
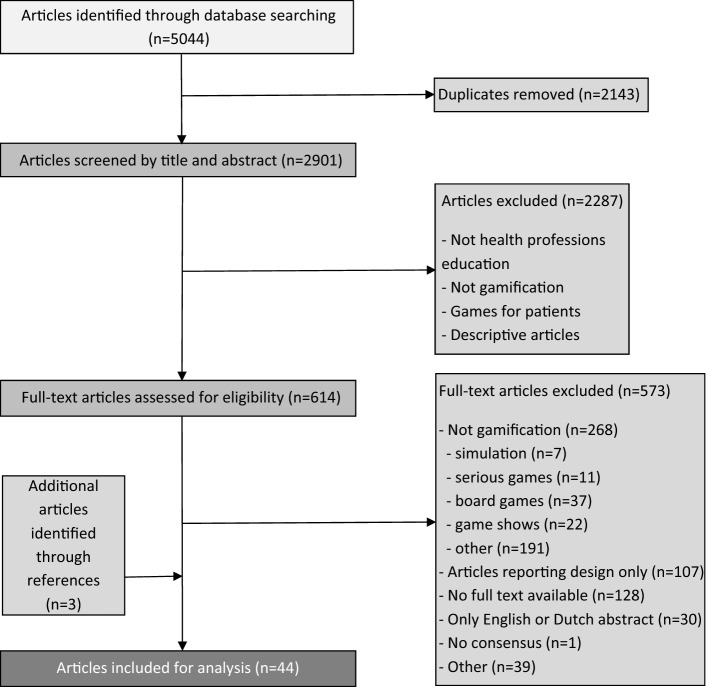
Flow chart of the article selection process

The study selection process is shown in Fig. 1. Our search identified 5044 articles, of which 38 met the inclusion criteria on the basis of full-text screening. Uncertainty about inclusion or exclusion of 20 other articles (Bigdeli and Kaufman 2017; Boysen et al. 2016; Campbell 1967; Courtier et al. 2016; Creutzfel dt et al. 2013; Dankbaar et al. 2016, 2017; Hudon et al. 2016; Inangil 2017; Kaylor 2016; Leach et al. 2016; Lim and Seet 2008; Mishori et al. 2017; Montrezor 2016; Patton et al. 2016; Richey Smith et al. 2016; Sabri et al. 2010) were resolved by consensus discussion among all members of the research team, which yielded three additional studies. Of the 17 studies excluded in this step, one was excluded because no consensus could be reached (Mullen 2018), the others did not meet the inclusion criteria. Hand search of references and citations yielded three additional studies. A total of 44 studies were eligible for inclusion in our systematic review (Table 1).

**Table 1:** Summary of gamified learning studies, categorized into purpose (description, justification, clarification)

Cat	Source	Population	Game attribute	Game-elements	Learning Focus	MERSQI score	Digital/analogue	Outcome
D (n = 25)	Ballon et al. (2004)	Residents (n = 173)	Assessment, human interaction	Puzzle, role play	Psychiatric diagnoses	6	Analogue	It was fun
	Bhaskar (2014)	Medical students (n = ?)	Assessment, environment, immersion, rules/goals	Achievements, missions	Blood groups	6	Digital/analogue	It was fun
	Chia (2013)	Nursing (n = 161)	Assessment, conflict/challenge, rules/goals	Avatars, levels, quiz, rewards, signposting	COPD treatment	7	Digital	Relevant for learning needs
	Davidson and Candy (2016)	Nursing (n = 30)	Assessment, rules/goals	Badges, levelling, quests, scoring	Evidence–based practice learning	7	Digital/analogue	High engagement and increased knowledge.
	Forni et al. (2017)	Medical students (n = 118)	Assessment	Puzzle	Biochemistry	6.5	Analogue	High satisfaction
	Henry et al. (2007)	Allied health students (n = 156)	Human-interaction	Role playing	Attitudes towards aging	13	Analogue	Improved attitudes
	Janssen et al. (2016)	Residents (n = 35)	Assessment, conflict/challenge, control	Scoring, competition, spaced-learning	Oncology	11.5	Digital	Overall satisfying and engaging
	Kalin (2016)	Medical students (n = 97)	Action language, assessment, conflict/challenge	Scoring, competition	Obstetrics	5	Digital	Students felt prepared
	Kerfoot et al. (2012)	Residents (n = 931)	Assessment, conflict/challenge, control	Scoring, competition, spaced learning,	Urology	12.5	Digital	Increased knowledge
	Kow et al. (2016)	Medical students (n = 221)	Action language, assessment, conflict/challenge	Scoring, competition	Patient safety	11.5	Digital	Helpful learning aid
	Lamb et al. (2017)	Residents (n = 46)	Assessment, conflict/challenge, human interaction	Scoring, competition, social network	General surgery learning	9.5	Digital	Increased use, enhanced knowledge
	Leach et al. (2016)	Specialists (n = ?)	Assessment	Awards	Prescription	8	Analogue	Increased knowledge
	Lin et al. (2015)	Medicine (mixed) (n = 49)	Assessment, conflict/challenge, environment, rules/goals	Levelling, scoring, time	Emergency medicine	7	Digital	Helpful learning aid
	Lobo et al. (2017)	Specialists (n = 16)	Assessment, conflict/challenge, human interaction	Scoring, competition, teams	Ultrasound training	7.5	Analogue	Overall satisfaction
	Longmuir (2014)	Medical students (n = 106)	Assessment	Scoring	Physiology	9	Digital	High satisfaction
	Mallon et al. (2016)	Specialists (n = 117)	Assessment, conflict/challenge, control	Rewards, competition, spaced-learning, teams	Guidelines	8	Digital	Overall satisfaction and increased knowledge
	Nemer et al. (2016)	Medical students (n = 79)	Assessment, conflict/challenge	Scoring, competition	Gynaecology clerkship preparation	8.5	Analogue	Enjoyable and educational
	Nevin et al. (2014)	Residents (n = 92)	Assessment, conflict/challenge	Badges, scoring, competition	Internal medicine	13	Digital	Highly engaging, increased knowledge
	Pacala et al. (2006)	Medical students (n = 1500)	Game-fiction, human interaction	Role playing	Enhancing attitudes towards elderly	6	Analogue	Overall satisfaction
	Pettit et al. (2015)	Osteopathic students (n = 106)	Assessment, conflict/challenge, control, human interaction,	Chance, scoring, mystery character, surprise, teams, time	Microbiology learning	7	Digital	Engaging
	Roubidoux et al. (2002)	Medical students (n = 42)	Assessment, conflict/challenge	Competition, scoring	Breast imaging	7	Digital	Overall satisfaction
	Snyder et al. (2013)	Residents (n = 169)	Assessment, conflict/challenge	Rewards, competition, surprise, time	Internal medicine	8	Digital	Overall satisfaction, increased knowledge
	Shah et al. (2010)	Pharmacy students (n = 172)	Assessment, rules/goals	Crossword puzzle	Pharmaceuticals	7.5	Analogue	Helpful learning aid
	Stanley and Latimer (2011)	Nursery (n = 96)	Assessment, game fiction, human interaction, rules/goals	Scoring, role playing, teams, time	Learning critical thinking	7	Analogue	Enjoyable
	Varkey et al. (2006)	Medical students (n = 84)	Control, environment, immersion	Role playing	Attitudes towards aging	9	Analogue	Increased attitudes towards aging
J (n = 12)	Adami and Cecchini (2014)	Medical students (n = 260)	Assessment, rules/goals	Crossword puzzle	Resuscitation principles	11	Analogue	Increased knowledge*
	Cook et al. (2012)	Nursing (n = 34)	Assessment, rules/goals	Scoring, Levelling, time, signposting	Life-support training	13.5	Digital	Enjoying but no difference in outcome*
	Van Dongen et al. (2008)	Residents (n = 22)	Action language, assessment, conflict/challenge, environment, immersion	Rewards, scoring, competition	Surgery (simulator)	10	Digital	Increased use
	El-Beheiry et al. (2017)	Residents (n = 49)	Action language, assessment, conflict/challenge, environment, immersion	Scoring, competition	Surgery (simulator)	13	Digital	Increased use, faster completion time
	Finley et al. (2012)	Medical students (n = 36)	Assessment, rules/goals	Scoring, levelling	Auscultatory skills	9	Digital	Increased knowledge*
	Kerfoot et al. (2014)	Primary care physicians (n = 111)	Action language, assessment, conflict/challenge, immersion	Rewards, scoring, competition,	Surgery (simulator)	9.5	Digital	Increased use
	Kerfoot et al. (2014)	Residents (n = 141)	Assessment, conflict/challenge, control	Scoring, competition, spaced-learning	Hypertension treatment	17	Digital	Increased use/knowledge, and changed prescription behaviour
	Lameris et al. (2015)	Medical students (n = 461)	Action language, assessment, conflict/challenge, rules/goals	Avatar, curiosity, rewards, time	Physiology	12	Digital	Increased use, enhanced knowledge*
	Rondon et al. (2013)	Speech-Language and Hearing Science (n = 29)	Assessment	Rewards	Anatomy & physiology	14.5	Digital	No difference with non-gamified version*
	Petrucci et al. (2015)	Residents (n = 14)	Conflict/challenge	Competition, progress, social network	Surgery (simulator)	11	Digital	Increased use, no difference in outcome
	Scales et al. (2016)	Residents (n = 422)	Assessment, conflict/challenge	Game-mechanics, rewards competition	Learning about quality improvement	12.5	Digital	Increased use, enhanced knowledge
	Worm and Buch (2014)	Medical students (n = 121)	Assessment, conflict/challenge	Scoring, competition	Biology	14.5	Digital	Increased use, enhanced knowledge
C (n = 7)	Butt et al. (2018)	Nursery (n = 20)	Assessment, conflict/challenge, immersion	Scoring, time	Urine catheterization	12	Digital	Highly engaged, no difference in outcome*
	Chen et al. (2017)	Medicine (mixed) (n = 60)	Assessment, conflict/challenge	Rewards (points and colours), time	Radiology	6	Digital	Helpful learning aid
	el Tantawi et al. (2018)	Dentistry (n = 98)	Assessment, conflict/challenge, game fiction, human interaction	Scoring, boss battles, competition, quests, story-telling	Scientific writing	14	Analogue	Modest satisfaction, increased knowledge
	Fleiszer et al. (1997)	Medicine (mixed) (n = 25)	Assessment	Award, quiz	Critical care surgery	7	Analogue	High satisfaction
	Koivisto et al. (2016)	Nursing (n = 166)	Assessment, conflict/challenge	Scoring, competition	Clinical reasoning	11	Digital	Overall satisfaction
	van Nuland et al. (2015)	Medical students (n = 67)	Assessment, conflict/challenge, human interaction	Scoring, competition	Anatomy learning	12	Digital	High motivation, increased knowledge
	Verkuyl et al. (2017)	Nursery (n = 47)	Assessment	Scoring	Paediatric knowledge	15.5	Digital	High satisfaction, increased knowledge*

The first column (Cat) contains categories for research purpose of gamification studies. Description studies (D) make no comparison, focus on observation and describe what was done. Justification (J) studies make comparisons between interventions, generally lack a conceptual framework or a predictive model and describe whether the new intervention worked. Clarification studies (C) apply a theoretical framework to understand and possibly explain the processes underlying the observed effects, describe why and how interventions (i.e. gamification) work and illuminate paths for future investigation. Question marks in study population indicates unknown or unmentioned number of participants. MERSQI = Medical Education Research Study Quality Instrument, * = confounded comparison group

As a form of triangulation and to assess the level of agreement between the researchers, a random sample of 10% of the excluded articles was double screened by the other members of the team. The overall agreement between JB, JSA, DJ and JRG was 94%. The disagreements about the remaining 6% were resolved by discussion, which led to the conclusion that all studies in the sample had rightly been excluded from the review.

### Educational context and student characteristics

The majority of the studies were conducted in the USA (n = 20) or Canada (n = 8). Most studies involved undergraduate (n = 15) and postgraduate medical students (e.g. residency) (n = 15), followed by nursing (n = 7), dental (n = 1), pharmacy (n = 1), osteopathic (n = 1), allied health (n = 1), speech language and hearing pathology students (n = 1) or a mix of students of different professional courses (n = 3) (Table 1). From the gamification studies in post-graduate medical education, the number of studies in surgical specialties (n = 6) equalled the number of studies in other medical specialties (n = 6). Compared to analogue gamified learning activities (n = 14), twice as many digital learning activities (n = 28) were identified. An example of gamification in a digital environment was a web-based platform where gamification elements were inspired by the Tetris game (Lin et al. 2015). The goal of that pilot study was to collect validity evidence for a gaming platform as training and assessment tool for surgical decision making of general residents. An example of an analogue gamified learning activity was the use of a board game for undergraduate medical students. The game consisted of a board depicting a mitochondrion and cards representing components of the mitochondrial electron transport chain that had to be put in the right order. The goal of that study was to assess the effect of this active learning activity (Forni et al. 2017).

### Game attributes

We categorized the identified game elements into the game attributes for learning of Landers’ framework (Landers 2014).

In most studies, the game attributes “assessment” (n = 40) and/or “conflict/challenge” (n = 27) (Table 1) were embedded in the learning environment. Intervention studies with assessment attributes particularly used scoring (n = 26) and rewards (n = 10). Scoring mostly entailed keeping record of points earned for completing a certain task or answering a question correctly. Rewards varied considerably and included digital trophies, donuts, iPads and money. Intervention studies with the conflict/challenge attribute particularly used competition (n = 21).

Combinations of game attributes were most common in our review (n = 36). Assessment and conflict/challenge attributes were often applied together (n = 24), predominantly in the form of leader boards displaying rank orders of participants, thus enabling comparison of students’ achievements. Compared to the other attributes, assessment attributes were more often examined separately (n = 6), followed by “conflict/challenge” (n = 1) and “human-interaction” (n = 1). The other attributes were always studied in combination with other game attributes (Table 1).

### Effects of gamified learning interventions

We did not find any negative outcome of the use of gamification in health professions education. All studies reported positive results compared to a control group not using gamification, or similar results for both groups (Table 1).

Multiple studies reported that the (frequently used) combination of assessment and conflict/challenge game attributes could increase the use of gamified learning materials (n = 8), strengthen satisfaction (n = 16) or improve learning outcomes (n = 11) (Table 1). Whether or not increased use ensured improved learning remained uncertain. For instance, two comparable studies using assessment and conflict/challenge attributes each reported increased use of simulators, but did not investigate or report learning outcomes (Kerfoot and Kissane 2014; Van Dongen et al. 2008). Two different studies in which the same gamified elements were used also found that the use of simulators had increased (El-Beheiry et al. 2017; Petrucci et al. 2015), but only one study found improved performance (El-Beheiry et al. 2017).

One study focused on the level of health care outcome (Kerfoot et al. 2014). This randomized controlled trial had the highest MERSQI score and investigated whether gamification in an online learning activity could improve primary care clinicians’ hypertension management. The intervention group participated in a gamified, spaced learning activity comprising three game elements: competition, space-learning and scoring. The control group received the same spaced education through online postings. The gamification intervention was associated with a modest reduction in numbers of days to reach the target blood pressure in a subgroup of already hypertensive patients. That study did not uncover the underlying mechanisms of how gamification supported these positive patient outcomes. A proper theory was also lacking or not presented. Because of the study design, we were not able to disentangle whether competition, spaced learning, scoring, or a combination of them had caused the effect. In fact, adopting and testing a combination of game elements without being able to disentangle their individual effects on learning is a quite general phenomenon in gamification research in health professions education.

### Contextual differences and effects of game attributes

We found that certain game attributes were more often applied to a particular context. For instance, a combination of conflict and challenge attributes was relatively more often applied to digital contexts (24 out of 28 digital studies) than to analogue contexts (3 out of 14 analogue studies). A combination of conflict and challenge attributes was also more often chosen by researchers from Europe (6 out of 8 European studies) and the USA (17 out of 21 USA studies) than by researchers from Canada (3 out of 8 Canadian studies). We found differences in use of a combination of conflict and challenge attributes between undergraduate and postgraduate settings (9 out of 15 undergraduate studies and 14 out of 15 postgraduate studies used such a combination). Yet, we could not find a direct indication that the effects of game attributes were dependent on these contextual factors.

### Quality of the current gamified learning research

The total MERSQI scores of the 44 studies included in our review ranged between 5 and 17 points (mean 9.8 points, SD 3.1; see Table 1).

#### Descriptive studies

Most of the included studies (n = 25; Table 1) were descriptive in nature in such sense that they focused on observation and described what was done, without using comparison groups. These descriptive studies were typically low in MERSQI scores (mean 8.3, SD 2.3) and only contained post-intervention measurements.

#### Justification studies

In twelve studies (Table 1), groups involved in gamified learning sessions were compared with control groups to investigate whether gamification enhances learning outcomes. Almost half of these justification studies were confounded in such a way that the outcomes could not be attributed to the gamification intervention under study, because the groups not only differed in treatment, but also with respect to other aspects (Table 1; marked with asterisks). Comparing a group of participants who took part in a gamified learning activity with a group of participants who did not take part in any learning activity is an example of a confounded comparison (Adami and Cecchini 2014). The remaining seven justification studies, which were without confounds, showed an average MERSQI score of 12.5 (SD 2.6), which was the highest study quality in our sample.

#### Clarification studies

In seven studies, theoretical assumptions were affirmed or refuted, based on the results of the study (Table 1). Three of these studies had a design with a control group (Butt et al. 2018; Van Nuland et al. 2015; Verkuyl et al. 2017), out of which two were confounded by poor design (Table 1; asterisks). The other four studies did not include control groups. With an average MERSQI score of 11.1 (SD 3.5), the seven clarification studies were of medium quality.

### The use of theory

The hallmark of clarification studies is the use of theory to explain the processes that underlie observed effects (Cook et al. 2008). In most studies (n = 5), multiple game attributes were related to a chosen theory. In three out of the seven clarification studies, the authors referred to Experiential Learning Theory (El Tantawi et al. 2018; Koivisto et al. 2016; Verkuyl et al. 2017) and in each of the remaining four studies the authors referred to a different theory: Reinforcement Learning Theory (Chen et al. 2017), Social Comparison Theory (Van Nuland et al. 2015), Self-Directed Learning (Fleiszer et al. 1997) and Deliberate Practice Theory (Butt et al. 2018). Each theory will be discussed briefly below, with specific attention to how these theories can be linked to game elements.

Experiential Learning Theory states that concrete experience provides information that serves as a basis for reflection. After this reflection, learners think of ways to improve themselves and, after this abstract conceptualization, they will try to improve their behaviours accordingly (Kolb et al. 2000; Kolb and Boyatzis 2001). Some researchers assumed that by gamifying their courses, students’ experiences and, consequently, their understanding (through reflection and conceptualization) might be enhanced. For instance, dentistry students’ experiences of being a part of an exciting narrative in an academic writing course, including game elements like role-playing, feedback, points, badges, leader boards and a clear storyline, were assumed to improve their performance (El Tantawi et al. 2018).

Self-Directed Learning is the process of diagnosing one’s own learning needs, formulating one’s own learning goals and planning one’s own learning trajectory. The increased autonomy in the pursuit of knowledge is assumed to result in higher motivation (Knowles 1980). Gamification that was inspired by Self-Directed Learning involved quizzes that were fully developed by small groups of medical students or residents about a self-chosen subject of their surgical intensive care unit rotation, right answers were rewarded with donuts (Fleiszer et al. 1997).

Deliberate Practice Theory is based on engaging already motivated students to become experts via well-defined goals, real world tasks and immediate and informative feedback (Butt et al. 2018; Ericsson et al. 1993). Deliberate Practice Theory was applied to develop an educational tool using game-elements (such as points and time constrains) and virtual reality to practice urinary catheterization in nursing education (Butt et al. 2018).

Two studies stood out for the way in which they used theory to explain the effect of a single game attribute. Van Nuland et al. (2015) used social comparison theory to explain the effect of competition and Chen et al. (2017) used Reinforcement Learning Theory to explain the effect of direct feedback in digital learning.

According to the Social Comparison Theory, social comparison is a fundamental mechanism for modifying judgment and behaviour through the inner drive individuals have to gain accurate self-evaluations (Corcoran et al. 2011; Festinger 1954). Gamification based on Social Comparison Theory involved the introduction of leader boards. According to Van Nuland et al. improved performance would be achieved by letting students identify discrepancies in their knowledge through upward comparison or validate their assumptions on knowledge through downward comparison (Van Nuland et al. 2015).

Reinforcement Learning Theory relates to a form of behavioural learning that is dependent on rewards and punishments (Börgers and Sarin 1997). If a desired behaviour or action is followed by a reward, individuals’ tendency to perform that action will increase. Punishment will decrease individuals’ tendency to perform that action. The gamification study based on this theory assumed that rewards and punishments (e.g. receiving points or negative, red-coloured responses, respectively) would improve the subjective learning experience and help learners acquire implicit skills in radiology (Chen et al. 2017).

Although different theories predicted the effectiveness of different mechanisms to improve performance, a common assumption seemed to be that the use of game attributes would improve learning outcomes by changing learning behaviours or attitudes towards learning.

## Discussion

The purpose of this systematic review was to investigate the current evidence for using gamification in health profession education and understand which mechanism are involved and how they can explain the observed effects.

The majority of the included studies—only quantitative and mixed-methods studies—were performed in medical schools in the USA and Canada, and used digital technologies to develop and implement gamified teaching and learning. No negative effects of using gamification were observed. Almost all interventions included assessment game attributes, mostly in combination with conflict/challenge attributes. Especially this combination of attributes was found to increase the use of learning materials, sometimes leading to improved learning outcomes. Our review revealed a relatively small number of studies involving high-quality control groups, which limited recommendations for evidence-based teaching practice. In addition, high-quality clarification studies on how underlying mechanisms could explain the observed effects are uncommon in gamified learning research. In most studies, an explicit theory of learning was not presented and an appropriate control group was lacking. Of the few studies that did refer to theory, most researchers essentially proposed that the game element(s) under study would strengthen attitudes or behaviours towards learning, which in turn might positively influence the learning outcomes.

### Empirical evidence for using gamification

At first glance, it may seem that improved or unchanged academic performance (e.g. learning behaviours, attitudes towards learning or learning outcomes) can justify the use of gamification in health professions education. However, caution should be taken in drawing strong conclusions, because most studies were descriptive, (confounded) justification or (confounded) clarification studies, or clarification studies that did not include control groups (total n = 36). In sum, despite the apparent encouraging early results, it remains unclear whether the reported effects on academic performance can be solely attributed to the gamified interventions due to the absence of (non-confounded) control-groups. The remaining eight studied included in our study were well-controlled studies using assessment and conflict/challenge attributes, so these study results could be interpreted with more confidence. The use of the learning material was increased in all intervention groups compared to their control groups (El-Beheiry et al. 2017; Kerfoot and Kissane 2014; Kerfoot et al. 2014; Petrucci et al. 2015; Scales et al. 2016; Van Dongen et al. 2008; Van Nuland et al. 2015; Worm and Buch 2014), often in combination with improved learning outcomes (El-Beheiry et al. 2017; Kerfoot et al. 2014; Scales et al. 2016; Van Nuland et al. 2015; Worm and Buch 2014). Using conflict/challenge and assessment attributes, especially competition and scoring, therefore seemed to positively influence learning. The apparently consistent effect of gamification is promising but also warrants further investigation. First, gamification research is still much in its infancy, it should be recognized that positive results may have been overreported due to a publication bias (Kerr et al. 1977; Møllerand and Jennions 2001) and that negative results remain un- or underreported. Second, so far, mainly small-scale and pilot studies have been conducted, which is not just typical of health professions education but also applies to areas where gamification is already more often used, such as computer sciences (Dicheva et al. 2015). Third, it is important to realise that gamification can also have unexpected or unwanted effects (Andrade et al. 2016). Competition, which is one of the most frequently used game elements in this review, is particularly interesting in that regard. In theory, competition can hamper learning by turning projects into a race to the finish line. In this case, participating in a gamified learning activity might diminish learning: winning becomes more important than the internalisation of knowledge and/or skills. This shift in attention from task to competition might, therefore, come at the expense of students’ performance and even their intrinsic motivation to learn (Reeve and Deci 1996). For example, in our review, four studies involving simulators showed that competition leads to increased use of the simulators (e.g., for a longer time and more frequently) (El-Beheiry et al. 2017; Kerfoot et al. 2014; Petrucci et al. 2015; Van Dongen et al. 2008). However, only one of these studies reported improved learning outcomes (El-Beheiry et al. 2017). That this outcome was not found in the other three studies might be attributed to a shift in attention as explained before. Increased use of learning material generally indicated repetition, which is one of the most powerful variables to affect memory, leading to improved learning outcomes and retention (Hintzman 1976; Kerfoot et al. 2009; Murre and Dros 2015; Slamecka and McElree 1983). However, as a corollary from using gamification in learning, repetition may become less effective when students’ attention shifts from the learning task to, for instance, competition. So even though repetition is vital for knowledge retention, increased repetition of the learning material in gamified interventions might not necessarily benefit learning, especially when students get distracted by game elements. Similarly, shifts of motivation may occur with different game attributes. In interventions applying the game attribute assessment, for example, the elements scoring and rewards are frequently used. However, giving rewards for a previously unrewarded activity can lead to a shift from intrinsic to extrinsic motivation and even loss of interest in the activity when the rewards are no longer given. This is also called the over-justification effect (Deci et al. 1999, 2001; Hanus and Fox 2015; Landers et al. 2015; Lepper et al. 1973). In such cases, students’ motivation might shift from being internally driven to learn to being externally driven by gamification, possibly ending with amotivation when the gamified activities are over.

We did not find a direct indication that the effects of game attributes were dependent on contextual factors, since all included studies reported positive results. However, we did find that a combination of conflict and challenge attributes was more often used in the context of postgraduate education and in digital modalities. Whether this implies that opting for digital modalities is better suited to postgraduate courses or whether digital modalities are better suited for a combination of conflict and challenge attributes remains uncertain. Future research should investigate whether other game attributes and/or modalities are also applicable to postgraduate students. In addition, researchers might focus on identifying reasons for choosing specific (combinations of) attributes in a specific context.

### The use of a conceptual framework

The conceptual framework we used in this study originated from serious games. It was altered by Landers (2014) for gamification purposes and was now—at least to our knowledge—used for the first time to systematically structure gamification studies. It was not the aim of this study to evaluate this method, however, future researchers may want to re-evaluate before applying it to systematic analyses. Coding was relatively easy which may imply that game-elements are over-generalized. For instance, points, badges, iPads and money are kinds of rewards and, therefore, confirmed as assessment attributes. However, the timing of the rewards (e.g., immediate versus delayed rewards) as well as the context of the rewards (e.g., negative or positive feedback) might have a different impact on the outcomes (Ashby and O’Brien 2007; Bermudez and Schultz 2014; Butler et al. 2007). Consequently, the claim that assessment attributes can increase or improve learning is insufficiently substantiated, or at least incomplete. Besides, some attributes appeared to have much overlap: immersion and environment attributes were almost always implemented in conjunction. This conceptual framework was helpful in guiding our review and interpreting the results, although some work is needed to refine its contents.

### Theory-driven gamification

The purpose of theory is to generate hypotheses, predict (learning) outcomes and explain underlying mechanisms. Unfortunately, most identified studies on gamification in health professions education were not based on theory, or theoretical considerations were not included or not yet elaborated. Our review showed that researchers who did use theory hypothesized that effective gamified learning might strengthen students’ learning behaviours or positive attitudes towards learning, which in turn might improve their learning outcomes. For instance, in studies referring to ELT (El Tantawi et al. 2018; Koivisto et al. 2016; Verkuyl et al. 2017), it was assumed that incorporating gamification into courses could enhance students’ experience and, therefore, their reflection on and conceptualization of that experience (Kolb et al. 2001). For example, El Tantawi et al. (2018) used story-telling and game-terminology (together with other game-elements) to improve students’ attitudes towards academic writing. This way, the researchers intended to modify students’ perceptions of a task and made it seem like an exciting adventure, with a story built around a fictitious organisation.

Next to changing attitudes, the aim of studies that applied theory was also to change behaviours. For example, reinforcement learning theory was applied to increase repetition by reinforcing right answers (Chen et al. 2017). Although all theories we identified in this review were useful in clarifying research findings in the field of gamification of education and learning, it remains difficult to explain on the basis of these theories why specific game attributes or combinations of them should be preferred over others. For instance, Van Nuland et al. (2015) used social comparison theory to develop a digital, competition-based learning environment and explain research outcomes. Social comparison theory poses that individuals compare their performances to those of others to evaluate their abilities and seek self-enhancement (Gruder 1971; Wills 1981). Van Nuland et al. (2015) aimed to trigger social comparison in an intervention group by using leader boards with peer-to-peer competition in a tournament environment. They found that the intervention group outperformed their noncompeting peers on the second term test. Here, the use of social comparison theory helped clarify this effect through the *comparative* element underlying the *competitive* features of gamified learning that may have increased participants’ motivation to excel. However, because scientific theories are general statements describing or explaining causes or effects of phenomena, it remains unclear which *specific* game element has the highest potential of triggering social comparison and whether competition should be the most viable option. Although the use of leader boards appears to be a valid choice, it can also hamper learning when it (1) shifts attention from learning to competition (see earlier), (2) is not liked by all students and (3) is not the only game attribute that triggers social comparison. Perhaps less competitive game elements (e.g., upgrading avatars, receiving badges, building things) or even different game attributes (e.g., control, game-fiction or human-interaction) could also trigger social comparison.

### Theoretical and practical implications

Based on the scarcity of high-quality studies on processes underlying the effects of gamified educational interventions, we urgently call for more high-quality clarification research. Clarification studies could provide researchers with an understanding of the mechanisms that are involved in gamified learning (and illuminate paths for future research) (Cook et al. 2008) and teachers with evidence-based information on how to implement gamification in a meaningful way. Based on our findings, future clarification studies should use and validate existing learning theories in the context of gamification. The theories we identified in this review could, among others, serve as a basis for this research (Landers 2014; Landers et al. 2015). Because theories are general ideas, researchers should focus on (separate) specific game elements to identify the most promising game attributes in relation to a specific theory. Important negative results should be reported as well. In addition, realist evaluation can help provide a deeper understanding by identifying what works for whom, in what circumstances, in what respects and how (Tilley and Pawson 2000). The finding that (a combination of) game attributes may enhance learning outcomes by strengthening learning behaviours and attitudes towards learning could be used as a starting point for such an approach.

In addition, we would like to emphasize the need for design-based research using well-defined controlled groups to find out whether gamified interventions work (justification research). Although justification research hardly allows for disentangling underlying processes, there should always be room for innovative ideas and interventions (e.g., applying infrequently used game attributes) to inform future research (Cook et al. 2008). Furthermore, design-based research on gamification can shed more light on learning outcomes. For instance, further research could illuminate whether increased repetition results in less positive learning outcomes when students’ attention is distracted from their learning task by one or more game attributes. We would also like to encourage design-based research for interventions that combine different outcome measures, such as learning outcomes and frequency of using gamified educational interventions.

Finally, most studies in this review showed promising results for implementing gamification in health professions education. This opens new ways for educators to carefully experiment with the way they teach and implement gamified learning in their curricula. First, they need to determine whether there are behavioural or attitudinal problems that need attention and can be resolved by integrating game elements into the non-gaming learning environment. Subsequently, they have to determine which game attribute or combination of game attributes and matching game elements may help prevent consolidation of undesirable behaviours and/or attitudes. In this sense, gamification could be seen as an experimental educational tool to resolve behavioural or attitudinal problems towards learning which, therefore, may improve learning outcomes.

### Strengths and limitations

The literature in this review represents a broad spectrum of gamified applications, investigated across the health professions education continuum. The strengths of our systematic review are the comprehensive search strategy using multiple databases, the use of explicit in- and exclusion criteria and the transparent approach to collecting data. Furthermore, our study offers a unique analysis approach implying a combination of four core elements—namely (a) an alternative way to distinguish gamification from other forms of game-based learning, (b) structuring game elements in a comprehensive way, (c) uncovering theories underpinning gamified intervention and (d) assessing study quality—which sets our study apart from existing systematic reviews on gamification. Using such an approach, we were better able to make a distinction between the three forms of game-based learning (gamification, serious games and simulations) and, therefore, to make an accurate selection of gamification studies. In doing so, we added a new perspective to literature reviews in health professions education by applying a conceptual framework and using a more generic way of structuring individual game elements into overarching game attributes to investigate whether there is a (combination of) game attribute(s) that holds the highest promise of improving health professions education.

This study had several limitations. First, although we took a systematic approach to identifying relevant articles, it is possible that we unintentionally overlooked some articles that explored the same phenomenon using different keywords. We also may have missed some articles while we had to exclude keywords that were too general and resulted in too many irrelevant articles. Yet, we tried to include as many relevant articles as possible by basing our search on a list of 52 game elements and Lander’s framework (Landers 2014; Marczewski 2017). We kept our search as comprehensive as possible while critically evaluating the output. In addition, we did not set a timeframe for our search since individual game elements were used in a non-game context long before the term “gamification” was coined.

Second, we are aware that other scholars may have different views of what constitutes (serious) games or gamified learning, since there is no consensus on the definition of “game”’ and, therefore, “gamification” and “serious games” (Arjoranta 2014; Ramsey 1923; Salen and Zimmerman 2004; Suits 1978). Since we explicitly added “game-intention” to our definitions of gamification, serious games and simulations, there may have been subjectivity in our decision-making process for inclusion/exclusion of studies. Views of designers, participating students, teachers and scholars can differ as to whether something is a game or not, because meanings are constructed on the basis of historical, cultural and social circumstances through specific discourse of games or acts of gameplay. This means that even though designers, researchers and teachers may have the intention to *not* create a game and to only use game-elements, a participants’ view of whether it actually *is* a game can be quite different, depending on his or her background. For instance, some students or researchers may interpret the inclusion of a leader board as a game element in a non-gaming context, while others may interpret it as a game. So, although the intention of creating a game is the characteristic difference between gamification and serious games, the interpretation of gamified interventions is prone to subjectivity due to a lack of consensus of what the word ‘game’ refers to. This, in turn, suggests that our study selection process may also be prone to subjectivity. However, the distinction we made between the three forms of game-based learning proved to be quite straightforward and we used a form of triangulation to overcome this limitation. Although we underline the need for clarification of terms like (serious) game and gamification, we do feel that our research method was appropriate for this study. A third limitation may be that only one researcher (AvG) was involved in screening the full texts to confirm the eligibility of each study on the basis of our in- and exclusion criteria. However, in case of uncertainties, the entire team was involved in the decision-making process and we undertook double screening on a 10% random sample of the excluded articles as a form of triangulation. Additionally, all researchers independently reviewed full texts during the process and engaged in joint discussions to resolve uncertainties and reach consensus, when necessary. The fourth limitation is that we included mixed methods studies, but ranked the included studies using MERSQI only. MERSQI is an instrument for assessing the methodological quality of experimental, quasi-experimental and observational studies in medical education, so it does not assess the qualitative parts of mixed methods studies. Although using the MERSQI enabled comparison between all studies included in our study, we realize that our outcomes neglected the quality of the qualitative parts of these studies and may not reflect the quality of each mixed-methods study in its entirety. We acknowledge, however, that the qualitative components of the mixed-methods studies may be very valuable. Fifth, although the applied conceptual framework enabled us to generalize our findings, at times, generalization might have caused too much information loss since the framework could use more refinement. Sixth and finally, we only included articles written in English and Dutch, so there is the potential for language and culture bias, since studies with positive results are more likely to be published in English-language journals (Egger et al. 1997).

## Conclusion

Gamification seems a promising tool to improve learning outcomes by strengthening learning behaviours and attitudes towards learning. Satisfaction rates are generally high and positive changes in behaviour and learning have been reported. However, most of the included studies were descriptive in nature and rarely explained what was meant by gamification and how it worked in health professions education. Consequently, the current research status is too limited to provide educators with evidence-based recommendations on when and how specific game elements should be applied. Future clarification research should explore theories that could explain positive or negative effects of gamified interventions with well-defined control groups in a longitudinal way. In this way, we can build on existing theories and gain a practical and comprehensive understanding of how to select the right game elements for the right educational context and the right type of student.

## Appendix

See Tables 1 and 2.

**Table 2:** Definitions and examples of gamified learning by attribute category

Attribute category	Definition	Example of gamified learning
Action language	The method and interface by which communication occurs between a player and the game itself	To participate in a learning activity, students are now required to use controls (e.g., a laparoscopic simulator)
Assessment	The method by which accomplishment and game progress are tracked	In a learning activity, points are used to track the number of correct answers obtained by each learner as each learner completes the activity
Conflict/challenge	The problems faced by players, including both the nature and difficulty of those problems	A small group discussion activity is augmented such that each small group competes for the “best” answer
Control	The degree to which players are able to alter the game, and the degree to which the game alters itself in response	A small group discussion activity is restructured such that each decision made by each small group influences the next topic that group will discuss
Environment	The representation of the physical surroundings of the player	A class meeting is moved from a physical classroom to a 3D virtual world (e.g. using virtual reality)
Game fiction	The fictional game world and story	Lectures, tests, and discussions are renamed adventures, monsters, and councils, respectively
Human interaction	The degree to which players interact with other players in both space and time	Learners participate in an activity where teams are used or interaction is needed to progress in that activity
Immersion	The affective and perceptual experience of a game	The gamified learning activity provide haptic feedback with realistic sound effects.
Rules/goals	Clearly defined rules, goals, and information on progress toward those goals, provided to the player	When completing worksheet assignments on tablet computers, a progress bar is displayed to indicate how much of the assignment has been completed.

Attribute categories were identified empirically by Bedwell et al. (2012), definitions were adapted from their work by landers (2015), examples were altered for the health professions education context
